# The association of membrane frizzled-related protein (*MFRP*) gene with acute angle-closure glaucoma – a pilot study

**Published:** 2008-09-08

**Authors:** I-Jong Wang, Shan Lin, Ting-Hsuan Chiang, Zoe Tzu-Yi Chen, Luke L.K. Lin, Por-Tying Hung, Yung-Feng Shih

**Affiliations:** 1 Department of Ophthalmology, National Taiwan University Hospital, College of Medicine, National Taiwan University, Taipei, Taiwan; 2 Department of Ophthalmology, School of Medicine University of California, San Francisco, CA; 3 Department of Ophthalmology, Taipei City Hospital Zhongxing Branch, Taipei, Taiwan

## Abstract

**Purpose:**

The membrane frizzled-related protein (MFRP) has been proposed as a probable candidate gene for extreme hyperopia and nanophthalmos, which are factors for angle-closure glaucoma. The purpose of our study was to investigate whether there are significant associations between angle-closure glaucoma and sequence variants in the *MFRP* gene reported previously in Taiwanese subjects.

**Methods:**

Genomic DNA was collected from 63 subjects with angle-closure glaucoma and 66 age-matched and gender-matched controls without angle-closure glaucoma. Three sequence variants were detected by polymerase chain reaction (PCR) and direct sequencing in all of the cases and controls.

**Results:**

None of the three sequence variants showed a significant result in terms of association with disease. The pairwise linkage disequilibrium (LD) mapping confirmed that these alleles have a comparatively strong LD index greater than 0.7 for D' and greater than 0.4 for r^2^ at these polymorphisms. However, we found there were no statistical associations between any of the three sequence variants located on *MFRP* and angle-closure glaucoma.

**Conclusions:**

In our pilot study, variations that we tested in *MFRP* were not associated with the development of acute angle-closure glaucoma in Taiwanese subjects.

## Introduction

Angle-closure glaucoma is an important cause of blindness in the Asian population particularly in those of Chinese and Taiwanese descent and may be acute, sub-acute, or chronic [1]. It has been established that primary angle-closure glaucoma (PACG) is associated with certain biometric ocular features such as shallow anterior chamber [2,3], increased thickness of the lens [4], and short axial length [5]. Such patients usually have a hyperopic refractive error [6]. The mechanisms involved in the pathophysiology and development of PACG are complicated and involve the anatomy of the anterior chamber angle as well as the spatial and anatomic relationships between the lens, iris, ciliary processes, and vitreous [7]. The pathophysiology of PACG usually involves an increase in lens thickness during the aging process, often in the setting of a relatively small eye and a shallow anterior chamber. By contrast, myopic eyes are typically longer and possess a thin sclera, particularly at the posterior pole. The human sclera of eyes with PACG and axial myopia undergo active remodeling and result in either excessive shortening or elongation of the axial length. Biochemical assays of highly myopic eyes show markedly reduced amounts of markers for collagen and glycosaminoglycans when compared with the similar region of sclera in emmetropic eyes [8].

Nanophthalmos, sometimes referred to as “simple (or pure) microphthalmos” [9], is a relatively rare condition characterized by a small eye in the absence of any systemic abnormalities. Nanophthalmic eyes show considerable thickening of both the choroidal vascular bed and scleral coat, which provide nutritive and structural support for the retina. The thickening of these tissues is a general feature of axial hyperopia whereas the opposite occurs in myopia. Both autosomal recessive and autosomal dominant inheritances have been reported for nanophthalmos [10,11]. The major clinical characteristics of the nanophthalmic eye include a short axial length, a high degree of hyperopia, a high lens/eye volume ratio, and a small corneal diameter [12,13]. The strong association of angle-closure glaucoma with nanophthalmos is thought to result from the ocular anatomic abnormalities seen in this condition [9,12].

The membrane frizzled-related protein gene (*MFRP*) is located on human chromosome 11q23.3 and is expressed predominantly in the retinal pigment epithelial cells and ciliary epithelial cells of the eye and at a lower level in the brain [14,15]. Recessive nanophthalmos is related to this unique locus, and four independent mutations in *MFRP* have been identified. This gene is not critical for retinal function as patients entirely lacking *MFRP* can still have good corrected vision, produce clinically normal electroretinograms, and show only modest anomalies in the dark adaptation of photoreceptors. *MFRP* appears primarily devoted to regulating axial length of the eye. It remains to be determined whether natural variation in its activity plays a role in any common refractive error.

In this report, we hypothesized that *MFRP* plays an important role in ocular axial length regulation of angle-closure glaucoma. We undertook a case-control study to identify potential sequence variants and their association with acute angle-closure glaucoma.

## Methods

### Patients

Unrelated Taiwanese subjects with acute PACG and unrelated control subjects with normal eyes were recruited at the National Taiwan University Hospital (Taipei, Taiwan) and Mackay Memorial Hospital (Taipei, Taiwan). All participants had similar social backgrounds and were from the local ethnic Han Chinese population with no ethnic subdivision. Informed consent was obtained from all subjects. This project had the approval of the institutional review board (IRB)/ethics committees of both hospitals and was performed in accordance with the World Medical Association's Declaration of Helsinki.

Patients were eligible for study participation if their acute PACG met the following diagnostic criteria: (1) the presence of at least two symptoms: eye pain, headache, blurred vision, and vomiting; (2) the presence of the following signs: conjunctival congestion, a mid-dilated unreactive pupil, and corneal edema; (3) 270 degree or greater of anterior chamber angle closure on gonioscopic examination; and (4) intraocular pressure (IOP)>40 mmHg by Perkins handheld applanation tonometry. All of these criteria were in compliance with the International Society for Geographical and Epidemiological Ophthalmology (ISGEO) classification of angle-closure glaucoma by Foster et al. [16]. Control subjects did not have a history of any of the aforementioned symptoms and signs. Every participant received a complete ocular examination, which included retinoscopy, slit-lamp evaluation of the anterior segment, measurement of intraocular pressure, axial length measurement (Sonomed Ultrasound A-1500; Sonomed Inc., Lake Success, NY), keratometry measurement, fundus examination, and detailed recording of the health and degree of cupping of the optic nerve head.

### DNA Extraction

After informed consent was obtained, 10-15 ml of venous blood was drawn from each participant, and total genomic DNA was extracted. DNA was purified from lymphocyte pellets according to described procedures using the Puregene kit (Gentra Systems, Minneapolis, MN) or the phenol-chloroform extraction method [17].

### Direct DNA sequencing

Screening was performed for possible mutations or single nucleotide polymorphisms (SNPs) in exons 4, 5, and 10 of *MFRP*, which have been previously studied to find the association with extreme hyperopia [18,19]. Exon 4 and exon 5 were amplified with a forward primer (AGG ACC CAG CTC CTC TGA AC) and reverse primer (CTT CCT CGG TTA GCC CTT CT). Exon 10 was amplified with another forward primer (AGG GCT GGT GCC CAG AAC AGC TGT CTG CTT T) and reverse primer (ATA CCT ACA CCC CCA GTA CCC CCA GAG TGT). Polymerase chain reactions (PCRs) were performed on 50 ng of genomic DNA with a GeneAmp PCR system 9700 thermocycler (Applied Biosystems, Foster City, CA) and correct temperatures for PCR. The PCR cycling conditions of exons 4 and 5 consisted of an initial denaturation for 2 min at 95 °C followed by 30 cycles of denaturation for 30 s at 95 °C, annealing for 30 s at 47.2 °C, extension for 40 s at 72 °C, and a final extension for 7 min at 72 °C. The PCR cycling conditions of exon 10 were an initial denaturation for 2 min at 95 °C followed by 35 cycles of denaturation for 30 s at 95 °C, annealing for 30 s at 52.3 °C, extension for 40 s at 72 °C, and a final extension for 7 min at 72 °C. Amplified PCR products were separated by agarose gel electrophoresis and visualized by staining with ethidium bromide. They were then purified using purification columns (QIAquick; Qiagen, Valencia, CA) and sequenced using dye terminator chemistry (BigDye Terminator version 3.1 on model 3100 Genetic Analyzer; Applied Biosystems, Foster City, CA). Sequences were trimmed for quality and aligned using BioEdit software (version 5.0.6. Copyright ©1997–2001 Tom Hall; Ibis Biosciences, Carlsbad, CA). Normal and affected individual DNA sequences were aligned to the known reference genomic sequence (NT_033899.7), which is available via the National Center for Biotechnology Information (NCBI) database, and compared for sequence variation.

### Statistical analysis

To examine the association of the sequence variants, the χ^2^ test was used to compare the alteration of genotypes between patients and control subjects. This analysis evaluates the difference between the observed genotype frequency and the expected frequency under the null hypothesis of no association. However, when the expected frequency is small, the Fisher exact test is conducted instead [20]. Bonferroni correction was applied for the multiple tests. Next, all detected sequence variants were assessed for Hardy–Weinberg disequilibrium using χ^2^ test [20]. To evaluate the effects of sequence variants on the risk of primary angle-closure glaucoma, we conducted logistic regression analysis with a stepwise approach [21]. The dependent variable was the disease status (patient, 1; control subject, 0), and the independent variables were values of sequence variants (homozygote, 2; heterozygote, 1; wild type, 0). The covariates of interactions between sequence variants were also included in the model. The final optimal model contains the statistically significant variables that were selected by the stepwise procedure. These statistical analyses were performed using SPSS software (ver. 10.1; SPSS Science, Chicago, IL). A p-value less than 0.05 was considered statistically significant.

The Haplo stat package in the software language R (version R 2.6.1) was used for haplotype analysis. Based on the haplotype structures, two linkage disequilibrium (LD) coefficients (Lewontin’s D′ and Hill’s r^2^) were obtained from the R package “genetics.” The Haploview program was applied to estimate pairwise LD between markers and partition haplotype blocks. A pair of SNPs is identified to have “strong LD” if the one-sided upper 95% CI boundary on D’ is greater than 0.98 and the lower boundary is greater than 0.7 [22].

## Results

In total, 63 patients with primary angle-closure glaucoma and 66 control subjects were enrolled in this study. There were 19 males and 44 females in the PACG group, and there were 42 males and 24 females in the control group. The mean age in the primary angle-closure glaucoma patients and control groups were 60.04±9.91 years and 59.12±6.75 years, respectively (p=0.547). The mean axial length was 23.37±0.75 mm (ranged from 21.57 mm to 25.21 mm) for right eyes and 23.32±0.76 mm (ranged from 21.35 mm to 24.63 mm) for left eyes of the control group (p=0.772). The mean axial length was 22.66±1.04 mm (ranged from 20.58 mm to 25.92 mm) for eyes with an attack and 22.59±1.09 mm (ranged from 20.63 mm to 26.03 mm) for fellow eyes in PACG subjects (p=0.731; Table 1).

**Table 1:** Axial lengths of subjects with acute PACG and control groups.

	**Axial Length (mm)**		
	**Attack eyes**	**Fellow eyes**	**Total eyes**
PACG Subjects	22.66±1.04	22.59±1.09	22.63±1.07
Range	20.58-25.92	20.63-26.03	20.58-26.03
Control Subjects			23.34±0.75
Range			21.57-25.21

The difference of axial lengths of attacked eyes in acute PACG groups (63 eyes) and mean axial lengths of control subjects (132 eyes) was tested statistically different with *t*-test and p value is 0.0001.

Only three sequence variants were identified within *MFRP* for all the cases and controls. Sequence variant rs3814762 (A/G) is located on exon 4 and leads to a nonsynonymous amino acid change from valine to methionine. Variants rs36015759 (T/C) and rs2510143 (C/T) are both located on exon 5 and result in synonymous changes.

Allelic frequencies of the three sequence variants in cases and controls are listed in Table 2. Although it was interesting that there was a trend toward association (p=0.06) for SNP rs36015759, no statistically significant association (χ^2^ test, p>0.015) was observed in this SNP. The subjects used in this study were justified by the Hardy–Weinberg’s exact test, and there is no genetic bias in any of the SNPs. We also evaluated the LD indexes for the specific LD block using three SNPs around the *MFRP* gene region. The pairwise LD mapping confirmed that these alleles have a comparatively strong LD index greater than 0.7 for D′ and greater than 0.4 for r^2^ (Table 3 and Table 4). However, there were still no statistically significant associations observed in these haplotypes.

**Table 2:** Sequence results of three SNPs in *MFRP*.

**Contig position**	**mRNA position**	**SNPrs**	**Nucleotide change**	**Location**	**AA change**	**Allele frequency**				**χ^2^**	**p**	**PACG**			**Control**			**χ^2^**	**p**
						**PACG**		**Control**				**n=63**			**n=66**				
22778647	553	rs3814762	A/G	Exon 4	Val to Met	G (0.794)	A (0.206)	G (0.735)	A (0.265)	1.2347	0.2665	GG (39)	GA (22)	AA (2)	GG (37)	GA (23)	AA (6)	1.2347	0.2665
22778695	639	rs36015759	T/C	Exon 5	Tyr to Tyr	C (0.722)	T (0.278)	C (0.818)	T (0.182)	3.3654	0.0666	CC (34)	CT (23)	TT (6)	CC (45)	CT (18)	TT (3)	3.3654	0.0666
22778971	687	rs2510143	C/T	Exon 5	His to His	T (0.135)	C (0.865)	T (0.106)	C (0.894)	0.5079	0.4761	TT (3)	TC (11)	CC (49)	TT (1)	TC (12)	CC (53)	0.5079	0.4761

Observed allele frequency of *MFRP* in cases and controls. AA change-amino acid change; SNPrs- public reference SNP number from the dbSNP database.

**Table 3:** Haplotypic analysis of the selected SNPs of *MFRP*.

**Haplotypes**			**Global score statistics**	**Haplotype specific test p value**	**Haplotype frequency**		**Odds ratio (with upper and lower limit of 95% CI)**
					**Control**	**Case**	
A	C	C	0.20396	0.2241	0.2247	0.1730	0.8778 (0.4479,1.7204)
G	C	C	0.20396	0.3312	0.5178	0.4143	1(NA)
G	C	T	0.20396	0.6709	0.0635	0.1016	1.0201 (0.4059, 2.5640)
A	C	T	0.20396	0.3228	0.0122	0.0333	3.9888 (0.3793, 41.9430)
G	T	C	0.20396	0.0428	0.1232	0.2778	1.9856 (1.0371, 3.8015)
A	T	C	0.20396	0.3569	0.0282	8.13x10^−8^	1.2188x10^−9^ (NA)
G	T	T	0.20396	NA	0.0303	NA	NA

**Table 4:** Pairwise linkage disequilibrium analysis between selected SNPs of *MFRP*.

		rs36015759	rs2510143
rs36015759	D		−0.0274
	D'		0.9982
	r		−0.2009
	χ^2^		10.4089
	p value		0.0013
	n		129
rs3814762	D	−0.0386	−0.0093
	D'	0.7138	0.3283
	r	−0.2163	−0.0675
	χ^2^	12.0687	1.1757
	p value	0.0005	0.2782
	n	129	129

D: Linkage disequilibrium estimate; D': Scaled linkage disequilibrium estimate; r: Correlation coefficient; χ^2^: χ^2^ statistic for linkage equilibrium; p value: χ^2^ p value for marker independence; n: Number of observations.

## Discussion

*MFRP* has been shown to be expressed in the retinal pigment epithelium (RPE) of the eye [14], which makes it a possible candidate gene for the regulation of axial length. In previous studies, we have shown that a retino-scleral signaling cascade may explain the changes in the scleral structure and composition during ocular axial length regulation [23,24] in which the RPE layer was proposed to transmit these signals [25].

Sundin et al. [18] have found an association between *MFRP* and nanophthalmos through a genetic linkage study. In their study, nanophthalmos was found to segregate with homozygous null mutations in *MFRP* while independent frameshift and stop codon mutations confirmed the association with nanophthalmos. *MFRP* null homozygotes frequently develop angle-closure glaucoma, cystoid macular edema, and serous retinal detachment, all conditions that are often related to hyperopia and microphthalmia [18]. Pauer et al. [26] have also observed clinical evidence of primary photoreceptor degeneration in *MFRP* null nanophthalmos patients. Aung et al. [27] found that *CHX10* and *MFRP* in Chinese subjects in Singapore are not associated with primary angle-closure glaucoma or with short axial length eyes.

In the present study, we found that *MFRP* revealed no sequence variants that would implicate this gene in the development of acute angle-closure glaucoma. In addition, our haplotype association study of SNPs on *MFRP* did not identify any significant association results for *MFRP* markers in these phenotypes. Our results indicate that variations in *MFRP* may not be a direct risk factor for acute angle-closure glaucoma. It may be that *MFRP* is involved in some cases of microphthalmia/anophthalmia but does not necessarily play a role in the more common disease of angle-closure glaucoma. Although the common link between the two disease states is a small eye, in the case of microphthalmia or anophthalmia, the defect is more extreme and present at birth.

The regulation of ocular axial length in patients who may be at risk for angle closure is likely a complex process in which other proteins may interact with *MFRP* and multiple genes contribute to the growth process. For example, the COOH-terminal domain of *MFRP* is known to be related to the Wnt-binding CRD (cysteine-rich domain) of the frizzled family of transmembrane proteins [28], and frizzled proteins are receptors for the Wnts, a family of cell–cell signaling molecules that mediate the regulation of growth, differentiation, and cell polarity during development [29]. Secreted Frizzled-related proteins, which contain homologs of the Wnt-binding CRD [30], are also thought to act as competitive inhibitors of Wnt signaling, and a similar function has been proposed for *MFRP* [15]. In this regard, we postulate that *MFRP* can work with other proteins to regulate the size of eyes, and specific sequence variations may not be necessary for this regulation.

In animal models, there are many reports addressing the role of *MFRP* in the development of the eye, but these functions may not be equivalent in humans. For example, the mouse mutant *rd6* has also been mapped and identified as a splice-site mutation in the orthologous *MFRP*, which is expressed in the retinal pigment epithelium (RPE) and ciliary body of the mouse eye [15]. The *rd6* mouse undergoes a slow degeneration of photoreceptors, and during this process, the fundus develops a distinctive array of depigmented spots that bear very striking resemblance to the human retinal degenerative disorders, Stargardt disease and fundus albipunctatus. Although many mutations in the mouse are known to cause microphthalmia, *rd6* mice have never been reported to have small eyes. This suggests that while the mouse depends more heavily on *MFRP* for the maintenance of photoreceptors, this gene is not used as part of a process of post-natal ocular growth since this process of secondary ocular growth appears to be vestigial. The small eyes in angle-closure glaucoma possess different anatomic features, which in most cases are not from a developmental defect of nanophthalmos and microphthalmos and their associated anatomic features such as the unusual thickness of the choroid and sclera [31].

The contents of the scleral coats will also likely play an important role in extreme nanophthalmos and angle-closure glaucoma. The sclera of the mammal is a typical fibrous connective tissue consisting primarily of collagen in which most of this collagen (as much as 99%) is type I [32]. However, low levels of other fibrillar collagen subtypes including type III, VI [33], XII [34], and V have been reported in the mammalian sclera [33]. In our previous study, we could not identify any associations of acute PACG with SNPs of these collagen-associated genes [35]. Proteoglycans are also a major component of the scleral ECM. The mammalian sclera is rich in hyaluronan, a unique, non-sulfated glycosaminoglycan that does not associate with a core protein of its own. Sclera also contains large amounts of dermatan and chondroitin sulfate-based proteoglycans [36], particularly the small proteoglycans, decorin and biglycan [37,38]. However, we also could not find any associations of keratocan, decorin, and DSPG3 with acute PACG. The amino acid changes from the SNPs of these proteoglycan genes are also not associated with the anatomic changes of acute PACG. Interestingly, our prior study revealed that SNP rs2664538, which is located within *MMP9*, is likely to be associated with acute PACG. We don’t fully understand the biological significance of this SNP in the development of acute PACG. Further study will be needed to identify additional potential candidate genes or haplotypes for the development of angle-closure glaucoma, which is strongly associated with small eye size.

In conclusion, sequence variants of *MFRP* do not appear to be associated with a risk for acute angle-closure glaucoma. Since our cohort of patients with an acute attack had significantly shorter eyes than the control group, our findings suggest that *MFRP* is not primarily and directly involved in ocular axial length regulation. However, there are still some significant limitations in our current study. Although there are a substantial number of patients in this pilot study, it is underpowered to statistically rule out a potential association. A multicenter study powered to have enough cases will be needed to adequately study these and other *MFRP* SNPs for their possible roles in the development of acute PACG. Second, since the Han-Taiwanese population was used in this study, the tagging SNPs in this group will be applied in a future multicenter study instead of using the SNPs of other populations. Because this was an exploratory pilot study, we studied established SNPs from previous publications, which showed biological significance. SNPs of *MFRP* have been searched on HapMap to find possible tag SNPs in the Han-Taiwanese population. There are six tag SNPs (rs948413, rs948414, rs10790289, rs3814762, rs883247, and rs883245), which can be found on HapMap. However, the SNPs genotyped in Chinese-Han are rs11217241, rs669462, rs848413, rs948414, rs12294677, rs10790289, rs12421909, rs2510143, rs3814762, rs4639950, rs883247, and rs883245 on HapMap. Among them, there are no sequence variants for rs12294677, rs669462, and rs4639950 in the Han-Chinese population. rs3814762 is the selected tag SNP from HapMap, which is also the SNP used in this study. However, there was no statistical difference of this SNP between “both groups” in our study. We plan to use appropriate tagging SNPs from HapMap and to genotype these variants in our future study. Furthermore, the tantalizing level of association (p=0.06) is very noteworthy, and it is of no major consequence that it is a synonymous variant. The importance is that it may actually be tagging a truly important functional variant. Future studies evaluating the relationship of other genetic regulators of axial length to *MFRP* may shed more light on its role in the complex cascade of axial length determination.
